# Heparan Sulfate Binding Cationic Peptides Restrict SARS-CoV-2 Entry

**DOI:** 10.3390/pathogens10070803

**Published:** 2021-06-24

**Authors:** Rahul K. Suryawanshi, Chandrashekhar D. Patil, Raghuram Koganti, Sudhanshu Kumar Singh, Joshua M. Ames, Deepak Shukla

**Affiliations:** 1 Department of Ophthalmology and Visual Sciences, University of Illinois at Chicago, Chicago, IL 60612, USA; rahuls@uic.edu (R.K.S.); cdpatil@uic.edu (C.D.P.); rkogan3@uic.edu (R.K.); sudhanshu2405@gmail.com (S.K.S.); james24@uic.edu (J.M.A.); 2 Department of Microbiology and Immunology, University of Illinois at Chicago, Chicago, IL 60612, USA

**Keywords:** SARS-CoV-2, pseudotyped virus, entry inhibitors, cationic peptide, heparan sulfate

## Abstract

A novel severe acute respiratory syndrome coronavirus 2 (SARS-CoV-2) has caused a global pandemic. While the world is striving for a treatment modality against SARS-CoV-2, our understanding about the virus entry mechanisms may help to design entry inhibitors, which may help to limit the virus spreading. Owing to the importance of cellular ACE2 and heparan sulfate in SARS-CoV-2 entry, we aimed to evaluate the efficacy of cationic G1 and G2 peptides in virus entry inhibition. In silico binding affinity studies revealed possible binding sites of G1 and G2 peptides on HS and ACE2, which are required for the spike–HS and spike–ACE2 interactions. Prophylactic treatment of G1 and G2 peptide was also proved to decrease the cell surface HS, an essential virus entry receptor. With these two mechanisms we confirm the possible use of cationic peptides to inhibit the entry of SARS-CoV-2.

## 1. Introduction

An outbreak of severe acute respiratory syndrome coronavirus 2 (SARS-CoV-2) occurred in Wuhan, China, in December of 2019 [1]. In the ensuing months, the virus has spread across the world and caused an ongoing global pandemic [2]. SARS-CoV-2 infections can develop into COVID-19, a respiratory illness that has an estimated mortality rate of 3.1% in the United States [3]. SARS-CoV-2 enters into host cells through the receptor-binding domain (RBD) of the spike (S) protein of the virus that must bind to the host angiotensin-converting enzyme 2 (ACE2) receptor. This interaction initiates fusion between the viral membrane and the plasma membrane, delivering the viral genome into the host cell [4]. 

Recent reports have implicated the cell surface glycosaminoglycan heparan sulfate (HS) as a key entry receptor for SARS-CoV-2 [4,5]. The spike protein is hypothesized to complex with both HS and ACE2 before membrane fusion can occur. Competitive inhibition of the HS–spike protein interaction using heparin inhibits viral entry and the subsequent infection [4,6]. Furthermore, treatment with heparin lyases, which cleave HS from the cell surface, also reduce viral infection [5]. The primary strain SARS-CoV also required HS to initiate entry [7]. Thus, emerging evidence suggests that HS could be a cellular target to treat SARS-CoV-2 infections.

Since HS contains many negatively charged sulfate groups, it binds to certain positively charged peptides with high affinity [8]. We have previously shown that two cationic peptides known as G1 (LRSRTKIIRIRH) and G2 (MPRRRRIRRRQK) bind to HS and inhibit viral entry of herpes simplex virus type 1 (HSV-1), another virus for which HS plays an essential role in entry [9,10]. G1 peptides possess alternating positively charged residues while G2 peptides have strings of cationic residues [9]. Here, we have explored G1 and G2 peptides as potential therapies to inhibit SARS-CoV-2 entry using a pseudotyped virus model. We have characterized the mechanism of action of the peptides and verified the interacting residue pairs of the peptides with the spike protein RBD.

## 2. Results

### 2.1. Docking Studies Revealed That G1 and G2 Peptides May Bind to SARS-CoV-2 and Inhibit Its Key Receptors

**Figure 1:**
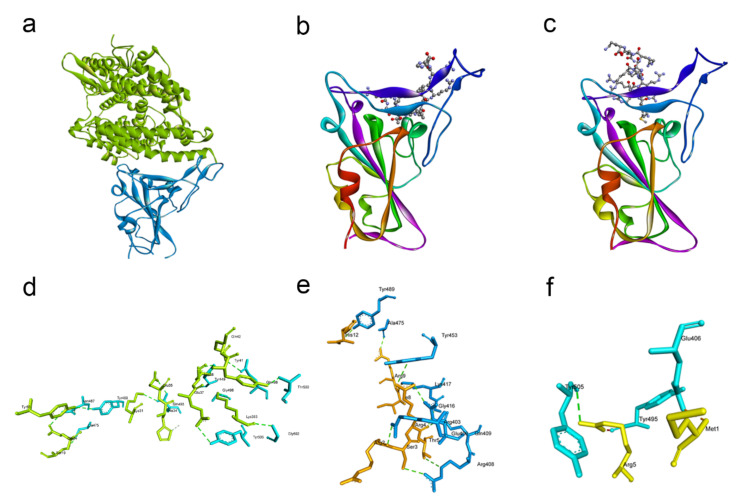
Molecular docking reveal the common residues involved in RBD–ACE2 and RBD–G1 peptide binding. (**a**) Structure of the spike RBD (in blue color) and ACE2 (in green color) complex. (**b**) Overall view of the G1 peptide (in ball-and-stick form) with the spike RBD (in cartoon form). (**c**) Full view of the G2 peptide (in ball-and-stick form) with the spike RBD (in cartoon form). (**d**) Interacting residues of ACE2 (green) with the spike RBD (blue). (**e**) Interacting residues of the G1 peptide with the spike RBD. (**f**) Interacting residues of the G2 peptide with the spike RBD.

We wanted to identify the key residues responsible for interaction between the receptor-binding domain (RBD) of the spike protein and the ACE2 receptor. Using the web server HPEPDOCK, we performed an in silico docking study of the spike RBD with ACE2 which revealed the most likely orientation by which the two proteins interact (Figure 1a). Next, we visualized the protein complex using Discovery Studio (Figure 1d) and identified the eight residue pairs involved in hydrogen bonds between the RBD and ACE2 (Table 1). We docked a peptide to the RBD and found that it binds to one of the aforementioned residue pairs, giving credence to the idea that the peptide could inhibit SARS-CoV-2 entry.

We then docked the G1 and G2 peptides with the spike RBD to check whether they could compeitively inhibit the spike–ACE2 interaction. Both peptides bound to the spike protein at similar sites (Figure 1b,c). Visualization of the peptide–spike protein interactions revealed that both the G1 and G2 peptides bound to at least one of the residues involved in the interaction of RBD with ACE2 (Figure 1e,f). The G1 peptide has ten residue pairs bound to the spike RBD (Table 2), and the TYR453 residue of the spike RBD can bind to both the ARG4 residue of G1 and the HIS34 residue of ACE2 (Table 1 and Table 2). Similarly, the G2 peptide has three residue pairs bound to the spike RBD, and the TYR505 residue of the spike RBD can bind to both the ARG5 residue of G2 and the GLU37 residue of ACE2 (Table 1 and Table 3). 

Given the affinity of the cationic peptides with the spike RBD shown through docking studies, we wanted to verify if the G1 and G2 peptides could bind to the other proteins on the surface of the virus. We obtained the structures of the E and M proteins through homology modeling and RCSB PDB, resepectively. Using HPEPDOCK again, we modeled the interactions of the peptides to the E and M proteins of SARS-CoV-2. Both the G1 and G2 peptides were predicted to form hydrogen bonds at multiple sites with the E and M proteins (Figures S1 and S2). The specific residues involved in each hydrogen bond are listed in Tables S1–S4. In addition, both peptides had significantly negative docking scores with each viral protein (Table S5). The G2 peptide in particular was predicted to bind more strongly to the E and M proteins than G1. As HPEPDOCK is not originally designed to dock channel proteins, we cannot conclude that the peptides bind to the E protein. However, the M protein interactions with both G1 and G2 are more probable. While docking studies are insufficient to conclude that the G1 and G2 peptides bind to the spike protein or the M protein, they spurred us to verify in vitro whether the peptides could inhibit viral entry. 

### 2.2. G1 and G2 Peptides Reduce Cell Surface Heparan Sulfate Expression

**Figure 2:**
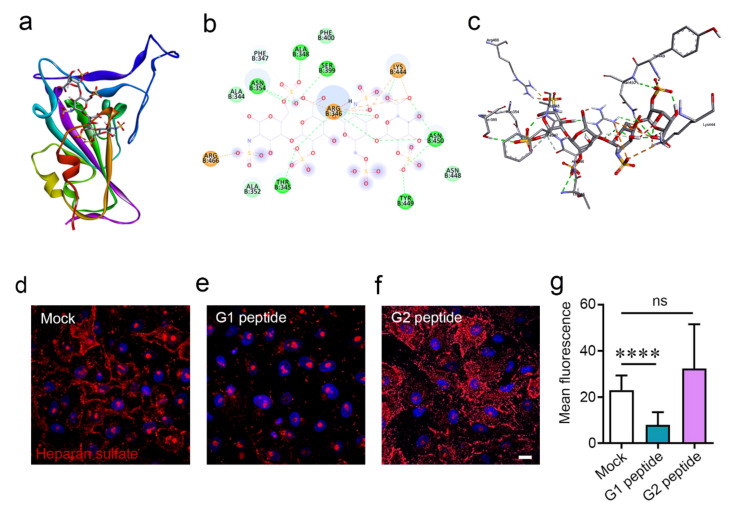
G1 and G2 peptides interact with heparin and causes downregulation of heparan sulfate on cell membrane. (**a**) Interaction of heparin (ball-and-stick form) with spike RBD (cartoon form). (**b**) Protein residues involved in the interaction of heparin with spike–RBD in 2D form. (**c**) Protein residues involved in the interaction of heparin (thick-stick form)with spike–RBD (thin-stick form) in 3D form. (**d**) Representative immunofluoresence images showing presence of heparan sulfate (red) on the cell surface in HEK cells treated with mock, (**e**) G1 peptide and (**f**) G2 peptide for 1 h. Blue color represents the nucleus stained with Hoechst stain. Scale bar = 10 μm. (**g**) Quantification of the surface heparan sulfate staining of d–f represented as the mean fluorescence. **** represents *p* < 0.0001.

Studies have implicated that HS is a key entry receptor for the SARS-CoV-2 spike protein [4]. To confirm these findings in silico, we docked the spike RBD to heparin, a glycosaminoglyan that is a close relative of heparan sulfate, using HPEPDOCK (Figure 2a). The spike protein formed nine bonding pairs with heparin (Figure 2b), and the resulting complex is visualized in Figure 2c. Heparan sulfate proteoglycans may be internalized by the host cell after binding to an appropriate ligand [11]. To understand the movement of the HS moieties after binding to the cationic peptides, we incubated cells with either the G1 peptide, G2 peptide, or a mock treatment for 1 h and stained for heparan sulfate using immunofluorescence microscopy. While the mock-treated group contained HS (red) located primarily on the cell surface (Figure 2d), the G1-treated cells lost the majority of their cell surface HS (Figure 2e). What little HS remains is found mainly in the nucleus (blue) of the cell, and as a small fraction of HS normally exists in the nucleus [12], it appears that the cell surface HS was shed from the cell. In contrast, treatment with the G2 peptide resulted in an internalization of HS into the cell cytoplasm (Figure 2f). Figure 2g represents quantification of the cell surface HS through a region-of-interest strategy. The results demonstrate that there is less HS on the cell surface of G1-treated cells compared to the mock treatment. For the G2-treated cells, the surface HS was not significantly different, which may be due to the technical challenge of differentiating the cell surface and intracellular HS as the HS was internalized in the cellular cytoplasm with G2 treatment (Figure 2f).

### 2.3. G1 and G2 Peptides Inhibit SARS-CoV-2 Pseudovirus Entry

**Figure 3:**
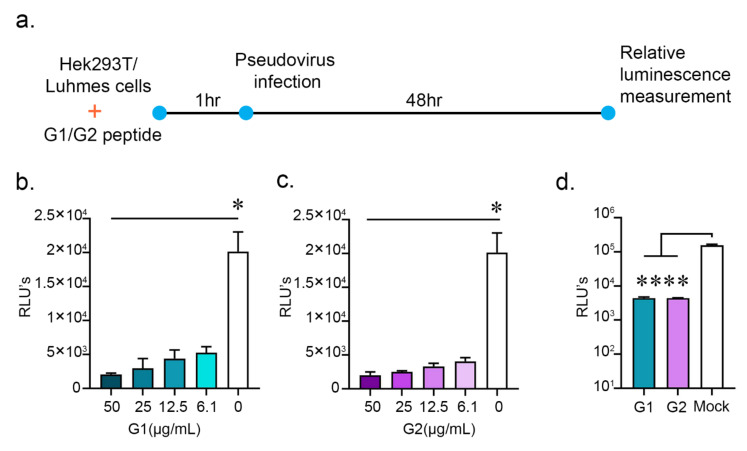
Cationic G1 and G2 peptides inhibit entry of pseudotyped SARS-CoV-2 particles in HEK and LUHMES cells. (**a**) Schematic showing prophylactic treatment of HEK293T and LUHMES with mock, G1 and G2 peptide followed by SARS-CoV-2 pseudotyped virus infection. The virus entry was estimated by relative luinescence units. (**b**) A graph showing entry of pseudotyped SARS-CoV-2 particles in HEK cells prophylactically treated with different concentrations of G1 peptide (μg/mL). (**c**) A graph showing entry of pseudotyped SARS-CoV-2 particles in HEK cells prophylactically treated with different concentrations of G2 peptide (μg/mL). (**d**) A graph showing pseudotyped SARS-CoV-2 particle entry into LUHMES cells after prophylactic treatment of G1 and G2 peptide at 50 μg/mL. * represents *p* < 0.05 and **** represents *p* < 0.0001.

We had observed that the G1 and G2 peptides may inhibit the spike RBD–ACE2 interactions and significantly reduce cell surface HS. SARS-CoV-2 has been recently reported to require heparan sulfate during entry [6]. To test whether the cationic peptides could inhibit the entry of the SARS-CoV-2, we generated a pseudovirus by transfecting (i) pCMV-MLVgagpol MLV gag and pol encoding plasmid and (ii) the pTG-Luc transfer vector with luciferase reporter with (iii) the spike plasmid (Supplementary Figure S1). We then incubated HEK cells with either G1 or G2 peptides at varying concentrations one hour prior to infection with the SARS-CoV-2 pseudotyped virus tagged with the luciferase enzyme (Figure 3a). At 48 h post infection (hpi), we lysed the HEK cells with cell lysis buffer and measured the luminescence of the samples with the help of the Promega luciferase assay system. A greater luminescence value, known as a relative light unit (RLU), would correspond to a greater degree of entry of the pseudovirus into the HEK cells. Prophylactic treatment with either the G1 or G2 peptides significantly reduced SARS-CoV-2 pseudovirus entry at concentrations ranging from 50 μg/mL to 6.1 μg /mL (Figure 3b,c). SARS-CoV-2 has also been reported to infect neuronal cells, causing symptoms including anosmia, ageusia, and seizures [13,14]. To investigate whether the G1 and G2 peptides could inhibit pseudotyped virus entry in a physiologically relevant neuronal cell type, we incubated the two peptides with Lund human mesencephalic (LUHMES) cells, 1 h prior to infection with the pseudotyped virus. At a concentration of 50 μg/mL, both peptides significantly reduced the pseudotyped virus’s entry into the LUHMES cells (Figure 3d). Figure S3 takes account of the cytotoxicity profile of the G1 and G2 peptides. With an MTT assay, the IC50 of the G1 peptides was found to be 1.3 mg/mL and that of the G2 peptide was 1.09 mg/mL. Looking at the IC50 values and active concentrations of the peptides, they may show very high selectivity indices, which is a very important aspect of any successful preclinical drug candidate.

## 3. Discussion

SARS-CoV-2 is known to interact with the cell surface receptors, including ACE2 and heparan sulfate glycoproteins, which serves as a primary entry receptor or facilitator of infection [4,15]. HS glycoproteins are ubiquitously expressed on the surface of many cell types and are involved in the infection of multiple viruses [13]. Targeting glycoproteins with the help of cationic peptides is a novel strategy with broad-spectrum application to inhibit viral adhesion to cell surfaces and subsequent entry and replication [9,15,16]. Apart from sequence-specific binding affinities, a negative charge on the cell surface glycoprotein plays an important role in virus–glycoproteins binding, thereby negatively charged peptides may interfere with virus–host interaction. With previous studies, we have demonstrated the efficacy of the G1 and G2 peptides in inhibiting entry of HSV-1 [9,10]. G1 and G2 peptides have been designed to bind to cellular HS at their 3-OS-HS (3-*O*-sulfated heparan sulfate) site. As emerging evidence implicates HS as an essential host receptor for SARS-CoV-2 entry, the G1 and G2 peptides may serve as antiviral therapies by reducing the cell surface HS present on host cells [4]. Currently, no standardized antiviral therapies for SARS-CoV-2 have been approved [17]. Interestingly, the G1 and G2 peptides appear to modulate HS expression through two different mechanisms despite only small differences in their structures. In addition to inhibiting the interaction of spike–HS, in silico studies have identified the TYR453 amino acid residue located on spike–RBD, which share binding affinities with both ACE2 and G1 peptide; thereby, we can speculate that G1 peptide may interfere with the interaction of spike–RBD and ACE2. Similarly, we accounted for the possible interaction of the G1 and G2 peptides with the other viral surface proteins E and M. Possibly due to their cationic nature, G1 and G2 peptides show a binding affinity with the E and M proteins at multiple sites. This phenomenon may be useful in hindering SARS-CoV-2 entry, but it would need to be tested in vitro.

SARS-CoV-2 has also been reported to infect neural progenitor cells [14]. To limit the spread of the virus in neuronal cells in addition to HEK293T cells, we show the efficacy of G1 and G2 peptides in LUHMES cells. Cytotoxicity studies on HEK293T cells indicate the non-toxic nature of the cationic peptides, which have also proved to be safe when used in in vivo studies for inhibiting the entry of herpesvirus at ocular sites [9,10].

While the experiments in this study were performed with a pseudotyped virus, we demonstrate that these cationic peptides may be suitable for further studies with the live virus SARS-CoV-2. Our findings add further evidence to the role of HS in SARS-CoV-2 infections; specifically, the binding specificity of the virus spike protein to the 3-OS-HS site and that inhibitors of the spike–HS interaction can reduce the potency of the virus. Given the ongoing pandemic, rapid development of an effective antiviral therapy remains a great scientific and public health priority.

## 4. Materials and Methods

### 4.1. Cell Line

Human embryonic kidney cells (HEK) cell line was purchased from ATCC (ATTCC CRL-1573 VA, USA). The cells were maintained in Dulbecco’s minimum essential medium (11955-065 Life Technologies, Carlsbad, CA, USA) supplemented with 10% fetal bovine serum (F2442, Sigma Aldrich, St. Louis, MO, USA) and 1% penicillin/streptomycin (15149-122, Life Technologies, Carlsbad, CA, USA). Lund human mesencephalic (LUHMES) cells (ATCC CRL 2927 VA, USA) were gifted from Dr. David Bloom’s lab and cultured as per Edwards and Bloom, 2019 [18]. Briefly, cells were cultured in proliferation medium with FGF (100-18B Peprotech, NJ USA) supplement for 72 h while changing the medium after every 24 h. Then, tetracycline (Sigma Aldrich, St. Louis, MO, USA) containing a differentiation medium was added and changed every 24 and 48 h for 4 days post incubation to induce the neuronal cell differentiation. Formation of neurite threads was confirmed under the microscope and through Western blot analysis with marker antibody (βIII-Tubulin TU-20 (4466S Cell Signaling Technology, Danvers, MA, USA) expression.

### 4.2. Peptide Synthesis and Preparation

The G1 (LRSRTKIIRIRH) and G2 (MPRRRRIRRRQK) peptides were synthesized at the Northwestern University Research Resources Center. The purity (>95%) and molecular weight of the peptide was confirmed by high-performance liquid chromatography and mass spectrometry, respectively. The G1 peptide was characterized on an Agilent 6520 Q-TOF LCMS (Agilent Technologies, Santa Clara, CA, USA) instrument using a 0–50% MeCN with 0.1% formic acid gradient from 5–30 min (the control was same instrument and solvent, but 5–95% gradient). The G2 peptide was characterized on a Waters Prep150 HPLC (Milford, MA, USA) using a 2–100% MeCN with 0.1% TFA gradient from 5–60 min. The G2 peptide solution was pH adjusted to pH 9 before injection. The working concentration of the peptides (10 mg/mL) was made in sterile PBS and was stored at −20 °C.

### 4.3. Cell Viability (MTT) Assay

An MTT assay was performed to analyze the toxicity of the different concentrations of the G1 and G2 peptides and their vehicle concentrations. A monolayer of HEK cells (96 well) was overlaid with different concentrations of G1 and G2 peptides and their vehicles, starting at 200 μg/mL, respectively. Phosphate buffer saline (at the vehicle concentration) served as a control for these experiments. Twenty-four hours post incubation, 5 μL of MTT reagent (2809, BioVision, Milpitas, CA, USA)) (5 mg/mL) was added to each reaction well followed by 3 h incubation at 37 °C in dark. Formed formazan crystals were dissolved in 50 μL acidified isopropanol (19516, igma Aldrich, St. Louis, MO, USA). After solubilization of the crystals, 40 μL of the supernatant was transferred to a new 96-well plate and measured at 550 nm and 630 nm optical densities. The reference wavelength was 630 nm. The percent viability was calculated using the following formula.

$$\mathrm{Percent}\;\mathrm{viability}=\frac{\mathrm{Mean}\;\mathrm{OD}\;\mathrm{of}\;\mathrm{treatment}}{\mathrm{Mean}\;\mathrm{OD}\;\mathrm{of}\;\mathrm{control}}\times 100$$

### 4.4. SARS-CoV-2 Pseudotyped Virus Particles Formation

Pseudotyped virus particles (SARS-COV2) were prepared as per Millet et al. (2019) [19]. Briefly, HEK293T cells were cultured in 6-well plates overnight to reach 40–50% confluency. The cells were further transfected with gag-pol plasmid (0.3 μg), luciferase-encoding plasmid (0.4 μg), and SARS-CoV-2 spike protein-encoding plasmid (0.3 μg) in Opti-MEM (31985-070, Life Technologies, Carlsbad, CA, USA). The culture plates were incubated at 37 °C with 5% CO_2_ in a cell culture incubator for 6 h and added with DMEM containing 10% FBS and 1% P/S and further incubated for 72 h. Pseudotyped viruses containing the cell supernatant were collected and filtered through a 0.45 μm filter to make the aliquots. The aliquots were stored at −80 °C until use (Figure S4a). The synthesis of pseudotyped SARS-CoV-2 particles was confirmed by entry assay (Figure S4b).

### 4.5. Entry Assay 

To check the virus neutralization potential of the G1 and G2 peptide on SARS-CoV-2 pseudotyped particles, the different concentrations of peptides (50–6.1 μg/mL) were added onto HEK cells and incubated for 1 h at 37 °C and 5% CO_2_. After incubation, the treated cells were infected with pseudotyped virus particles for two hours and the media was replaced by DMEM containing 10% FBS and 1% P/S and incubated for 48 h. After incubation, the pseudotyped virus entry was evaluated by measuring chemiluminescence by a luminometer (BioTek synergy H1, Winooski, VT, USA). The Promega luciferase assay system (E1500, Promega, Madison, WI, USA) was used to get the relative luminescence units.

### 4.6. Heparan Sulfate Labelling

HeK cell cultures were performed in glass bottom dishes (P35G-1.5-20-C, MatTek Ashland, MA, USA). The cells were treated with mock (phosphate buffer saline), G1, or G2 peptide. After 1 h of incubation, the cells were gently washed with PBS and fixed by 4% paraformaldehyde for 10 min. Cells were again washed and blocked using 1% bovine serum albumin (BSA) (A2153, Sigma Aldrich, St. Louis, MO, USA) at room temperature. After 1 h incubation, the cells were incubated with anti-heparan sulfate antibody conjugated with fluorophore (F58-10E4 clone cat no. 370255S, AMSBIO, Cambridge, MA, USA) and made in BSA. The Hoechst stain (R37605, Invitrogen, Waltham, MA, USA) was used to stain nuclei. After 1 h incubation, the cells were gently washed with PBS and resuspended in PBS. The cells were imaged under LSM 710 (Carl Zeiss, GmbH, Germany) confocal microscopy at 63× magnification. Image analysis was performed using ZEN 3.1 software (Carl Zeiss, GmbH, Germany). The cell surface HS was quantified in terms of mean fluorescence using the region-of-interest strategy.

### 4.7. Homology Modeling

The sequence of the SARS-CoV-2 envelope (E) protein was obtained from UniProt (accession ID: A0A6B9WFC7). BLASTp on the E protein was performed using the PDB database as a search set. The top result of the BLASTp search (PDB ID: 5X29 [20]) was used as a template for homology modeling using the GalaxyWEB server [21]. The model validation was performed using molprobity [22,23] and the ProSA webserver [24,25] (Figure S5). These validations confirmed the quality of the model, allowing it to be used in docking studies (Table S6).

### 4.8. Docking Studies

The experimentally determined structures of the SARS-CoV-2 spike RBD complexed with its receptor ACE2 were downloaded from the RCSB PBD (PBD ID: 6VW1) [26]. The structure of the main protease or M protein was downloaded from the RCSB PBD as well (PBD ID: 6LU7). The structure of the E protein was obtained using homology modeling. Nonrelevant chains, water, and ligands were deleted from the PBD files. Interactions between the residues of the RBD and ACE2 were visualized using BIOVIA Discovery Studio Visualizer. Docking of the G1 or G2 peptides to the spike RBD, E, and M proteins was performed using the web server HPEPDOCK [27]. The spike RBD was docked with heparin using the heparin mode on ClusPro [28]. The top 10 docking results were analyzed, and a subset of the models were visualized using BIOVIA Discovery Studio Visualizer.

### 4.9. Statistics Analysis

Error bars of all figures represent the mean and SD of three independent experiments (*n* = 3). The experimental dataset between the groups was compared using ordinary two-way ANOVA and two-tailed unpaired Student’s t-tests. Differences between values were considered significant when * *p* < 0.05, ** *p* < 0.01, *** *p* < 0.001, and **** *p* < 0.0001.

## Figures and Tables

**Table 1:** Residues involved in the hydrogen bond between the spike RBD and ACE2 receptor.

Spike RBD Residue Involved in the Hydrogen Bond	ACE2 Residue Involved in the Hydrogen Bond
GLN493	LYS31
THR500	TYR41
GLN498	GLN42
TYR449	ASP38
ASN487	GLN24
GLN493	GLU35
TYR505	GLU37
TYR453	HIS34
ALA475	SER19
ASN487	TYR83
TYR489	TYR83
GLY496	LYS353
GLY502	LYS353

**Table 2:** Residues involved in the hydrogen bond between the spike RBD and G1 peptide.

Spike RBD Residue Involved in the Hydrogen Bond	G1 Peptide Residue Involved in the Hydrogen Bond
GLU406	ARG4
ARG403	ARG2
ARG408	SER3
LYS417	ILE8
TYR489	HIS12
ALA475	ARG9
ARG408	THR5
GLY416	ARG4
TYR453	ARG4
GLN409	ARG4

**Table 3:** Residues involved in the hydrogen bond between the spike RBD and G2 peptide.

Spike RBD Residue Involved in the Hydrogen Bond	G2 Peptide Residue Involved in the Hydrogen Bond
GLU406	MET1
TYR505	ARG5
TYR495	ARG5

## Supplementary Materials

The following are available online at https://www.mdpi.com/article/10.3390/pathogens10070803/s1, Figure S1. Molecular docking of the G1 and G2 peptides with the E protein. (a) Structure of the SARS-CoV-2 E protein (cartoon) and the G1 peptide (ball-and-stick) complex. (b) Visualization of the interacting residues between the E protein and the G1 peptide. (c) Structure of the SARS-CoV-2 E protein (cartoon) and the G2 peptide (ball-and-stick) complex. (d) Visualization of the interacting residues between the E protein and the G2 peptide.Figure S2. Figure S2. Molecular docking of the G1 and G2 peptides with the M protein. (a) Structure of the SARS-CoV-2 M protein (cartoon) and the G1 peptide (ball-and-stick) complex. (b) Visualization of the interacting residues between the M protein and the G1 peptide. (c) Structure of the SARS-CoV-2 M protein (cartoon) and the G2 peptide (ball-and-stick) complex. (d) Visualization of the interacting residues between the M protein and the G2 peptide. Figure S3. G1 and G2 peptides are non-toxic at active concentrations. An MTT assay showing the percent viability of HEK293T cells at different concentrations of (a) G1 and (b) G2 peptide. Figure S4. (a) Pseudotyped virus production. A combination of pCMV-MLV gag and pol encoding plasmid, pTG-Luc transfer vector with luciferase reporter, and the SARS-CoV-2 spike plasmid were co-expressed in HEK cells. The plasmid concentrations were used according to Millet et al. (2019). (b) Validation of pseudotyped virus particles was performed by entry assay, showing relative luminescence units as a measure of pseudotyped virus entry. The spike from SARS-CoV and VSV was used as control. Figure S5. Results of modeling the E protein using the ProSA webserver. The overall model quality Z-score = −0.41. Table S1. Residues involved in the hydrogen bond between the E protein and G1 peptide.Table S2. Residues involved in the hydrogen bond between the E protein and G2 peptide. Table S3. Residues involved in the hydrogen bond between the M protein and G1 peptide. Table S4. Residues involved in the hydrogen bond between the M protein and G2 peptide. Table S5. Docking scores for various protein–ligand complexes. The E–G1, E–G2, M–G1, and M–G2 complexes are scored below. Table S6. Summary of the homology modeling validations. The MolProbity score, Ramachandran Favored Regions, and the ProSA-web server Z-score are provided.
