# Supplementary material for: Heparan Sulfate Binding Cationic Peptides Restrict SARS-CoV-2 Entry
Source: Pathogens. 2021 Jun 24;10(7):803. doi: 10.3390/pathogens10070803 (PMC8308704; doi:10.3390/pathogens10070803)
Supplement: Supplementary file 1 [file pathogens-10-00803-s001.zip › pathogens-1256150-supplementary.pdf]

Supplementary document

Heparan Sulfate Binding Cationic Peptides Restrict SARS-CoV-2 Entry

Rahul K. Suryawanshi [1], Chandrashekhar D. Patil [1], Raghuram Koganti [1], Sudhanshu Kumar Singh [1], Joshua M. Ames [1] and Deepak Shukla [1,2,*]

[1] Department of Ophthalmology and Visual Sciences, University of Illinois at Chicago, Chicago, IL 60612, USA; rahuls@uic.edu (R.K.S.); cdpatil@uic.edu (C.D.P.); rkogan3@uic.edu (R.K.);
sudhanshu2405@gmail.com (S.K.S.); james24@uic.edu (J.M.A.)

[2] Department of Microbiology and Immunology, University of Illinois at Chicago, Chicago, IL 60612, USA

* Correspondence: dshukla@uic.edu

Abstract: A novel severe acute respiratory syndrome coronavirus 2 (SARS-CoV-2) has caused a global pandemic. While the world is striving for a treatment modality against SARS-CoV-2, our understanding about the virus entry mechanisms may help to design entry inhibitors, which may help to limit the virus spreading. Owing to the importance of cellular ACE2 and heparan sulfate in SARS-CoV-2 entry, we aimed to evaluate the efficacy of cationic G1 and G2 peptides in virus entry inhibition. In silico binding affinity studies revealed possible binding sites of G1 and G2 peptides on HS and ACE2, which are required for the spike–HS and spike–ACE2 interactions. Prophylactic treatment of G1 and G2 peptide was also proved to decrease the cell surface HS, an essential virus entry receptor. With these two mechanisms we confirm the possible use of cationic peptides to inhibit the entry of SARS-CoV-2.

Keywords: SARS-CoV-2; pseudotyped virus; entry inhibitors; cationic peptide; heparan sulfate

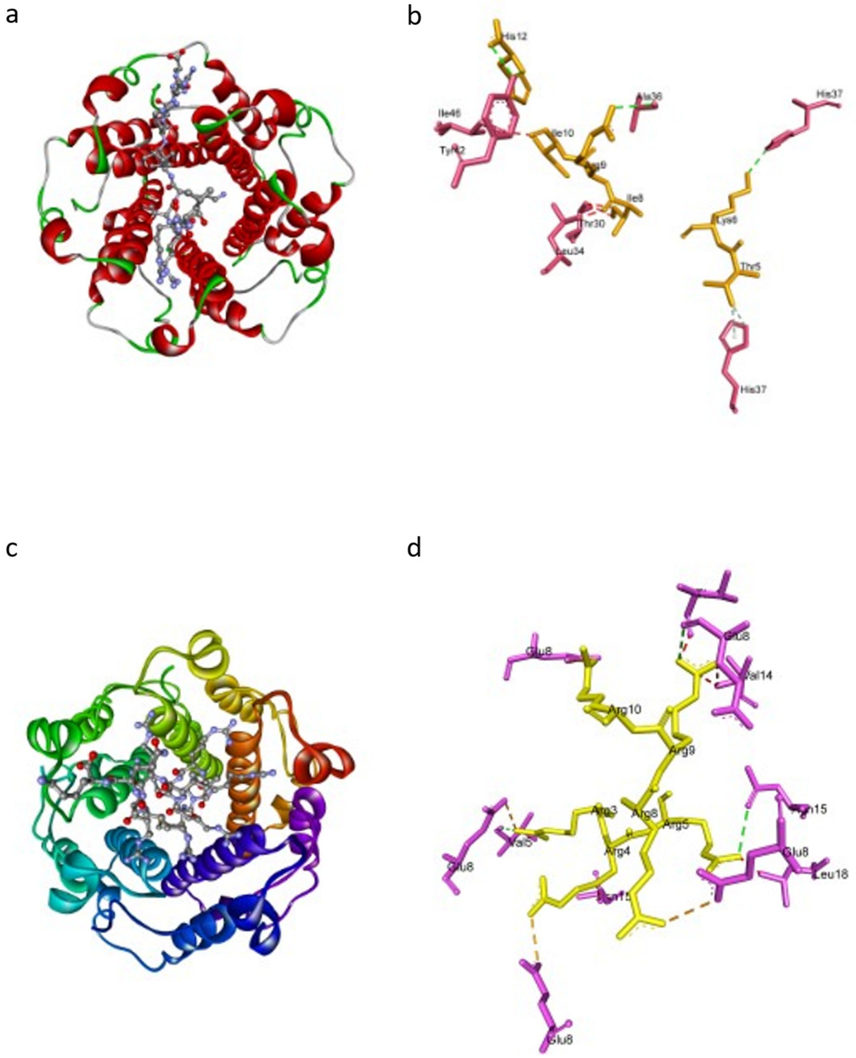

Figure S1. Molecular docking of the G1 and G2 peptides with the E protein. (**a**) Structure of the SARS-CoV-2 E protein (cartoon) and the G1 peptide (ball-and-stick) complex. (**b**) Visualization of the interacting residues between the E protein and the G1 peptide. (**c**) Structure of the SARS-CoV-2 E protein (cartoon) and the G2 peptide (ball-and-stick) complex. (**d**) Visualization of the interacting residues between the E protein and the G2 peptide.

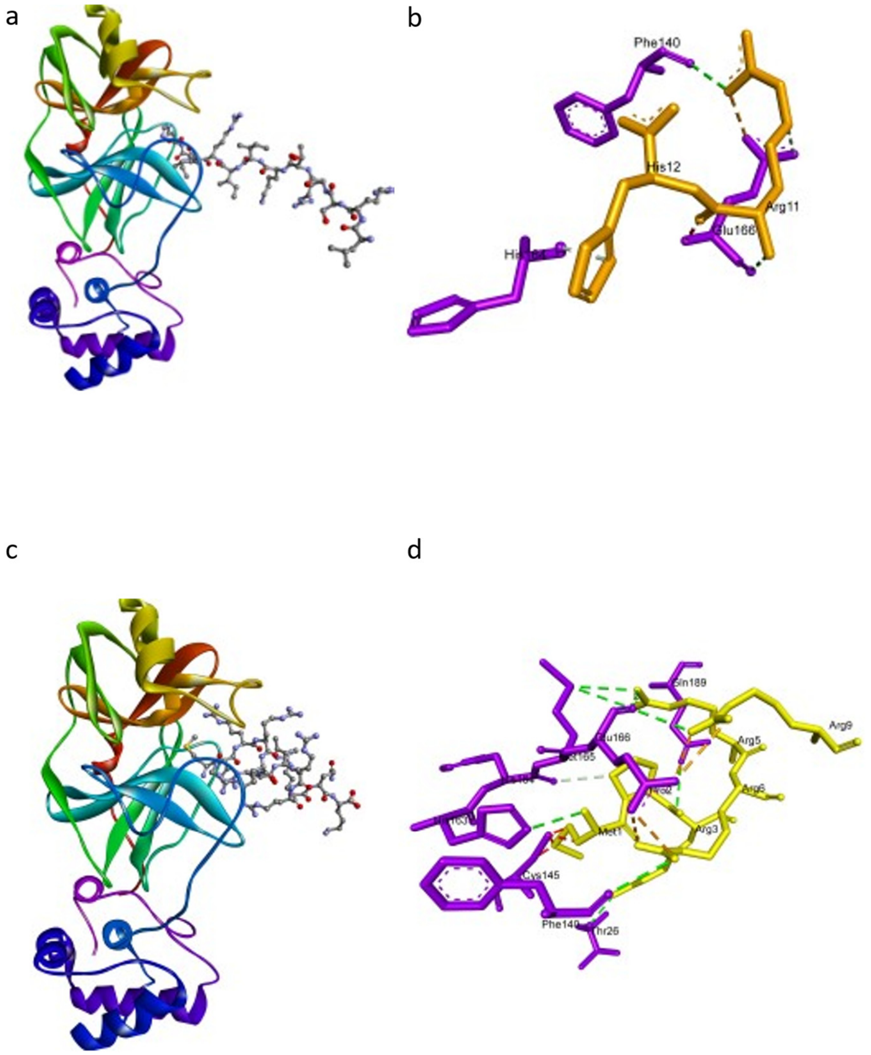

Figure S2. Molecular docking of the G1 and G2 peptides with the M protein. (**a**) Structure of the SARS-CoV-2 M protein (cartoon) and the G1 peptide (ball-and-stick) complex. (**b**) Visualization of the interacting residues between the M protein and the G1 peptide. (**c**) Structure of the SARS-CoV-2 M protein (cartoon) and the G2 peptide (ball-and-stick) complex. (**d**) Visualization of the interacting residues between the M protein and the G2 peptide.

Table S1. Residues involved in the hydrogen bond between the E protein and G1 peptide.

E Protein Residue Involved in the Hydrogen Bond	G1 Residue Involved in the Hydrogen Bond
Chain A: THR30	ILE8

Chain A: LEU34	ILE8
Chain A: ILE46	ILE10
Chain A: TYR42	HIS12
Chain C: HIS37	LYS6
Chain B: ALA36	ARG9
Chain E: HIS37	THR5

Table S2. Residues involved in the hydrogen bond between the E protein and G2 peptide.

E Protein Residue Involved in the Hydrogen Bond	G2 Residue Involved in the Hydrogen Bond
Chain A: VAL5	ARG3
Chain A: GLU8	ARG3
Chain A: ASN15	ARG4
Chain B: GLU8	ARG4
Chain C: LEU18	ARG5
Chain C: GLU8	ARG8
Chain C: ASN15	ARG5
Chain D: VAL14	ARG9
Chain D: THR11	ARG9
Chain D: GLU8	ARG9
Chain E: GLU8	ARG10

Table S3. Residues involved in the hydrogen bond between the M protein and G1 peptide.

M Protein Residue Involved in the Hydrogen Bond	G1 Residue Involved in the Hydrogen Bond
GLU166	ARG11
PHE140	ARG11
HIS164	HIS12

Table S4. Residues involved in the hydrogen bond between the M protein and G2 peptide.

M Protein Residue Involved in the Hydrogen Bond	G2 Residue Involved in the Hydrogen Bond
CYS145	MET1
GLU166	ARG6
GLU166	ARG9
GLU166	ARG5
MET165	ARG5
HIS163	MET1
PHE140	ARG6
GLN189	ARG3
THR26	ARG3
HIS164	PRO2

Table S5. Docking scores for various protein–ligand complexes. The E–G1, E–G2, M–G1, and M–G2 complexes are scored below.

Protein-Ligand Complex	Docking Score
E protein with G1 peptide	−210.749
E protein with G2 peptide	−213.981
M protein with G1 peptide	−193.291
M protein with G2 peptide	−213.638

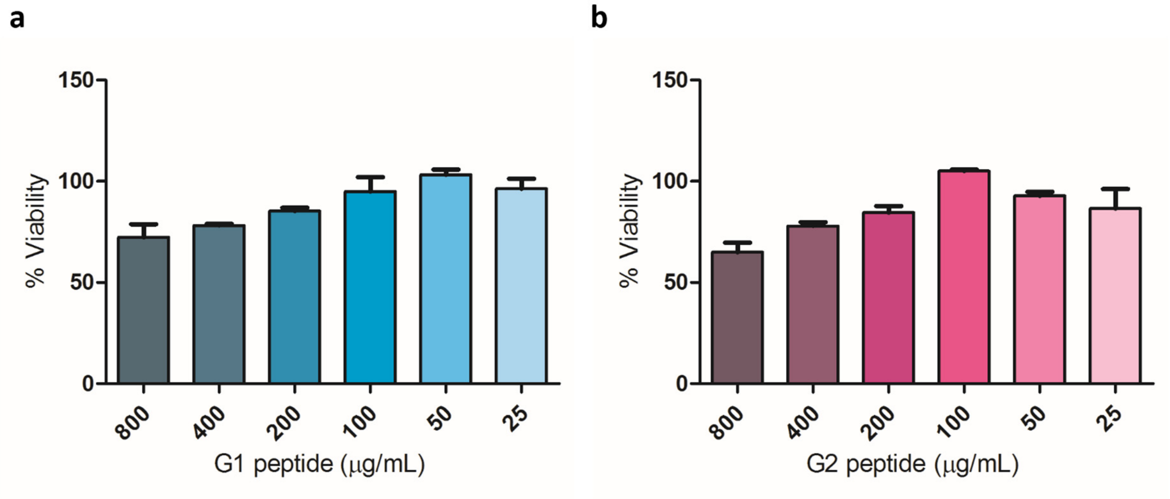

Figure S3. G1 and G2 peptides are non-toxic at active concentrations. An MTT assay showing the percent viability of HEK293T cells at different concentrations of (**a**) G1 and (**b**) G2 peptide.

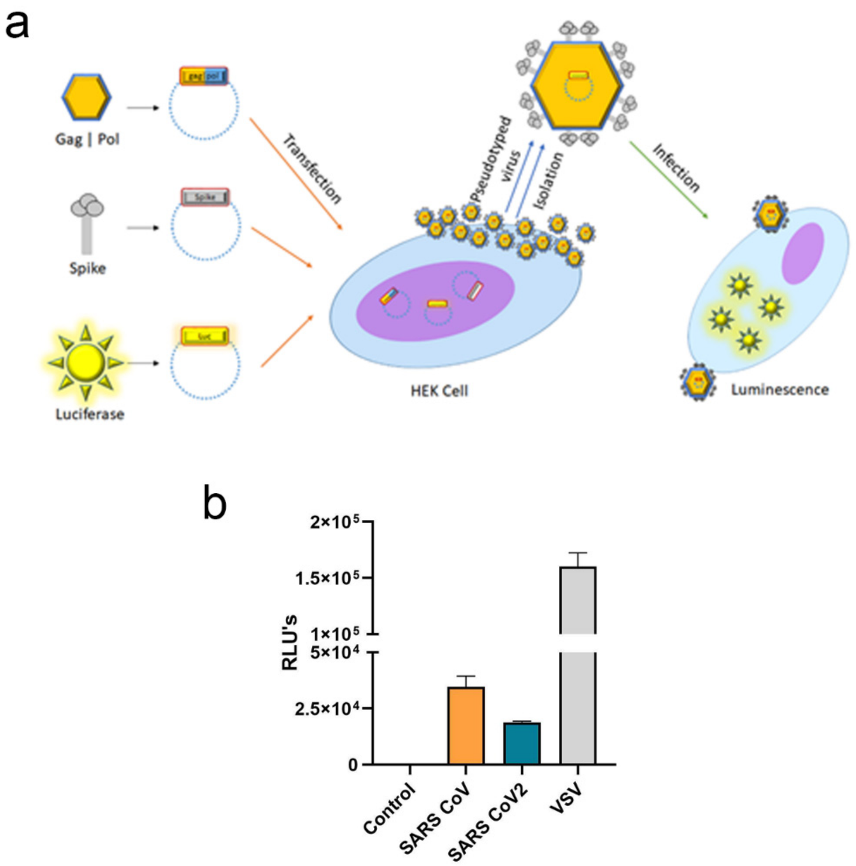

Figure S4. (**a**) Pseudotyped virus production. A combination of pCMV-MLV gag and pol encoding plasmid, pTG-Luc transfer vector with luciferase reporter, and the SARS-CoV-2 spike plasmid were co-expressed in HEK cells. The plasmid concentrations were used according to Millet et al. (2019). (**b**) Validation of pseudotyped virus particles was performed by entry assay, showing relative luminescence units as a measure of pseudotyped virus entry. The spike from SARS-CoV and VSV was used as control.

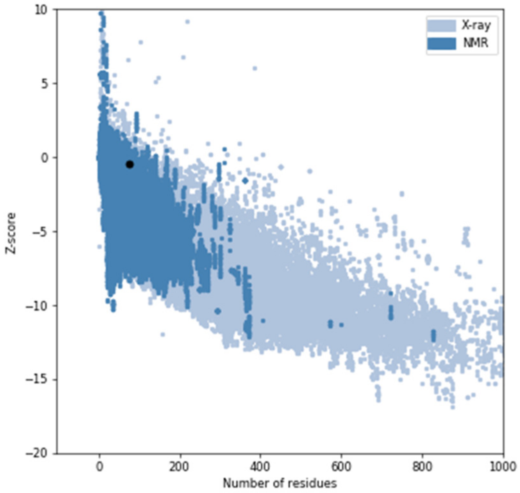

Figure S5. Results of modeling the E protein using the ProSA webserver. The overall model quality Z-score = −0.41.

Table S6. Summary of the homology modeling validations. The MolProbity score, Ramachandran Favored Regions, and the ProSA-web server Z-score are provided.

**Name**	**MolProbity Score**	**Ramachandran Favored Regions**	**ProSA-Web Server Z-Score**
E-protein of SARS-CoV-2	2.67	95.89%	−0.41
